# The BRCA1/2 pathway prevents hematologic cancers in addition to breast and ovarian cancers

**DOI:** 10.1186/1471-2407-7-152

**Published:** 2007-08-06

**Authors:** Bernard Friedenson

**Affiliations:** 1 Department of Biochemistry and Molecular Genetics, College of Medicine, University of Illinois Chicago, 900 South Ashland Ave, Chicago, IL 60607, USA

## Abstract

**Background:**

The present study was designed to test the hypothesis that inactivation of virtually any component within the pathway containing the BRCA1 and BRCA2 proteins would increase the risks for lymphomas and leukemias. In people who do not have BRCA1 or BRCA2 gene mutations, the encoded proteins prevent breast/ovarian cancer. However BRCA1 and BRCA2 proteins have multiple functions including participating in a pathway that mediates repair of DNA double strand breaks by error-free methods. Inactivation of BRCA1, BRCA2 or any other critical protein within this "BRCA pathway" due to a gene mutation should inactivate this error-free repair process. DNA fragments produced by double strand breaks are then left to non-specific processes that rejoin them without regard for preserving normal gene regulation or function, so rearrangements of DNA segments are more likely. These kinds of rearrangements are typically associated with some lymphomas and leukemias.

**Methods:**

Literature searches produced about 2500 epidemiology and basic science articles related to the BRCA pathway. These articles were reviewed and copied to a database to facilitate access. Meta-analyses of statistical information compared risks for hematologic cancers vs. mutations for the components in a model pathway containing BRCA1/2 gene products.

**Results:**

Deleterious mutations of genes encoding proteins virtually anywhere within the BRCA pathway increased risks up to nearly 2000 fold for certain leukemias and lymphomas. Cancers with large increases in risk included mantle cell lymphoma, acute myeloid leukemia, acute lymphocytic leukemia, chronic lymphocytic leukemia, and prolymphocytic leukemia. Mantle cell lymphoma is defined by a characteristic rearrangement of DNA fragments interchanged between chromosomes 11 and 14. DNA translocations or rearrangements also occur in significant percentages of the other cancers.

**Conclusion:**

An important function of the BRCA pathway is to prevent a subgroup of human leukemias and lymphomas that may involve non-random, characteristic gene rearrangements. Here, the genetic defect in BRCA pathway deficiencies is a chromosomal misrepair syndrome that may facilitate this subgroup of somatic cancers. Inactivation of a single gene within the pathway can increase risks for multiple cancers and inactivation of a different gene in the same pathway may have similar effects. The results presented here may have clinical implications for surveillance and therapy.

## Background

BRCA1 and BRCA2 proteins are thought to be essential to prevent breast/ovarian cancer largely because of the high lifetime risks faced by carriers of mutations in the corresponding genes. More modest increases in risk for other cancers have also been noted [1-5]. Basic science studies find multiple biologic functions for BRCA1 and BRCA2 proteins [6-15], including participating within a pathway that mediates error-free repair of DNA double strand breaks by homologous recombination [15].

**Figure 1:**
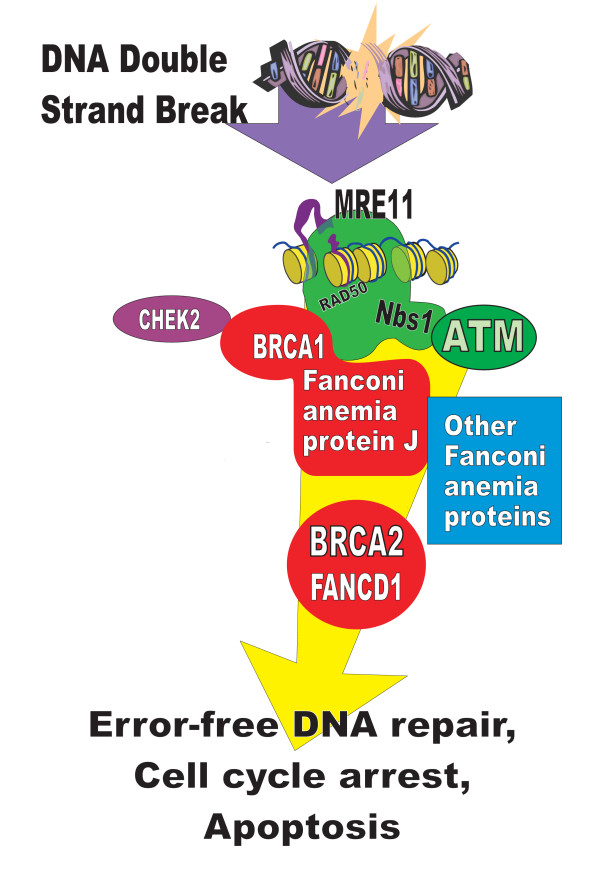
Schematic model for the "error-free" BRCA double strand break repair pathway Brief overview of components within the BRCA pathway used here as a working model that was tested here. The model is based largely on reference 16. BRCA2 is the same as FANCD1 and the interaction between BRCA1 and Fanconi anemia protein J is shown. While the gene products shown represent the over-all pathway, "error-free" double strand break repair by homologous recombination undoubtedly involves other proteins but the discussion is limited to those shown. Not shown are details of the 13 Fanconi anemia gene products and additional components including EMSY, a whole family of RAD51 related proteins, DCC, cohesins and accessory proteins. Deficiency states may be rare or unknown for these additional proteins and large epidemiologic studies are uncommon. Other protein kinases related to ATM carry out similar functions in response to other genotoxic stresses, and some of them collaborate with ATM. Proteins within the pathway also interact with other branches of the DNA damage response and with further proteins.

Fig. 1 summarizes a model for this error-free double strand break repair pathway (based on reference [16]). BRCA1 and BRCA2 gene products are placed within a sequence encompassing the MRE11, Rad50 and NBS1 complex (MRN complex), ATM, CHEK2, BRCA1, BRCA2, and Fanconi anemia proteins. For the purposes of this paper, this model will be referred to as the "BRCA pathway."

A critical protein function lost from anywhere within this error-free repair BRCA pathway may force repair of DNA double strand breaks by lower fidelity, error prone methods. Risks for cancers mediated by such errors should then greatly increase. Lymphomas and leukemias can be associated with large gene rearrangements, which can be pictured as arbitrary rejoining of broken DNA fragments. For example, almost all mantle cell lymphomas have a characteristic interchange between pieces of chromosomes 11 and 14 [t(11;14)(q13;q32)]. In some leukemias, the make-up of a fusion protein may bear witness to other abnormal repairs [e.g. [17]]. Error-tolerant repair may also leave other signs such as in the acute myeloid leukemias, where there may be evidence of abnormal gene fusions, duplications, inversions, deletions or reciprocal translocations [18]. The present study was designed to test the hypothesis that inactivation of a critical component of the BRCA pathway would favor gene rearrangements that underlie some lymphomas and leukemias. The results show that risks for a subset of leukemias and lymphomas increase up to nearly 2000 fold. The results may have clinical implications for surveillance and chemotherapy.

## Methods

The study was designed to review the risks for leukemias and lymphomas associated with a deleterious mutation within a prototype BRCA pathway (Fig. 1) for error-free double strand break repair. The purpose of this article is not to examine functionality of specific gene variants, but rather to examine the effect of loss of gene function anywhere within a testable pathway on risk for specific hematologic cancers. For many of the studies examined, especially case series, the exact genetic variant is unknown but loss of gene function (regardless of the reason) was confirmed by other means (e.g. RNA, protein or other tests). Genes examined were ATM, NBS1, MRE11, BRCA1, BRCA2, Fanconi anemia genes usually studied as a group including 13 known genes, and CHEK2.

PubMed, PubMed Central, Google, and Google scholar searches were conducted to collect relevant research articles related to the model BRCA pathway. These searches were for epidemiology studies published within the last ~20 years and basic science articles published within the past ~15 years. About 2500 articles were reviewed and copied to a database to facilitate search and further review. Where available statistical information permitted, meta-analysis with heterogeneity testing was conducted comparing cancer risk vs. deleterious mutation of a pathway gene.

Case-control, cohort and prevalence studies were reviewed. Data from studies that measured cancer incidences associated with epigenetic modification of pathway genes and/or alterations in protein or mRNA levels were also included. Epidemiologic studies were excluded in whole or in part if they did not provide required data or permit calculation of required information or if they were superseded or subsequently invalidated. The rarity of mutations in some molecules limited data available and limited the analysis of some BRCA pathway components. As far as possible, statistical analyses were limited to gene variants either known to eradicate normal protein function or to severely lower normal levels. All the mutations were spontaneously occurring and/or inherited except for therapy related (somatic) inactivation of BRCA1 in acute myeloid leukemia (AML). To verify that therapy related disease did not bias the results it was compared to data for primary AML.

Epidemiologic data was tabulated as odds ratios or relative risks: for ATM associations with NHL as MCL, with ALL, CLL, and PLL; for Fanconi anemia gene associations (13 known genes) with primary AML, with leukemia before age 15 and with ALL; for BRCA1 associations with primary and therapy related AML and with CML; for BRCA2 associations with AML, ALL and CLL; for NBS1 associations with lymphomas, ALL and NHL; and for CHEK 2 associations with CLL.

The DerSimonian-Laird random effects model[21] was used throughout since it relaxes the assumption of a common effect due to mutation. This may be more appropriate here than fixed effects models since inactivating mutations can in theory have different targets with different effect sizes. However, the uncertainty bounds for random effects are more conservative and often larger. When at least three studies were available, meta-analysis was performed. For the 9 studies available for ATM mutations in MCL, potential methodological confounders were ruled out by generating subgroups without the potential confounder. Statistical associations were compared to independent basic science experiments and to basic science theory.

Data from the NCI Surveillance Epidemiology and End Result (SEER) program was used to compare incidences in the general population for the approximate age ranges in Fanconi anemia groups. The NCI "DevCan" program was used to calculate cumulative control incidences for cancers affecting Fanconi anemia patients. Population data in DevCan came from 10,000 patients using 1999–2001 figures, but was matched as closely as possible to patient numbers in the Fanconi anemia study. Relative risks and confidence intervals for random effects models were then calculated by StatsDirect and RevMan. In Fanconi anemia, death, bone marrow transplant, AML and solid tumors censor or alter the natural history of other conditions but competing risk models were not used.

The prevalence of ATM mutation heterozygotes in the general population is widely cited as 0.3% to 1%. The incidence of biallelic mutations which are required to cause the hereditary disease A-T is much smaller (3/million to 11/million) [22,23]. Use of any value within this range as a control would give much larger risks. However, a population prevalence of 1% for ATM mutations was used to prevent overstating differences from the general population.

Heterogeneity was calculated as non-combinability of odds ratios by the Breslow-Day method, from the inconsistency statistic[24], by a moment based method and graphically from L'Abbe plots. None of the meta-analyses presented showed evidence for heterogeneity by these criteria. Chi-square tests on combined odds ratios were performed. Bias was assessed using the method of Egger and by inspecting funnel plots for asymmetry[25]. There was no statistical evidence of publication bias for summary estimates (results not shown).

A general limitation of meta-analysis is that access to original data is limited or the data is so old that some calculations in publications are impossible to reproduce. Fortunately, some articles used in meta-analyses contained both raw and final calculated data. This enabled control experiments to check the validity of calculations based on raw data. Testing raw instead of final data gave no or very small errors as confirmed dozens of times. To rule out computer program errors, the RevMan program from the Cochrane Review Group was used to verify some calculations made by StatsDirect. Microsoft Excel with the data-analysis add-in was used for some calculations.

## Results

The present study was designed to test the hypothesis that inactivation of virtually any component within the BRCA pathway would increase risks for lymphomas and leukemias. Risks were summarized [see Additional file 1] for leukemias and lymphomas vs. mutations or aberrations at numerous steps within the model pathway in Figure 1. Each of the genes within the BRCA pathway is considered below. The conclusion emerges that inactivation of any of these genes greatly increases risks for a subgroup of leukemias and lymphomas. This subgroup includes (B-cell) mantle cell lymphoma (MCL), acute myeloid leukemia (AML), T-cell acute lymphocytic leukemia (T-ALL), chronic lymphocytic leukemia (B-CLL) and T-cell prolymphocytic leukemia (T-PLL). The large increases in risk suggest that preventing these diseases must be an important physiologic function of the complete BRCA pathway. The results also suggest a mechanism for this function.

### Inactivation of the BRCA pathway gene ATM favors a translocation associated with mantle cell lymphoma

9 studies of the incidence of ATM mutations in MCL from a total of 363 patient samples were summarized [see Additional file 1]. Meta-analysis seemed appropriate initially because all the studies found very strong odds ratios for an ATM-MCL association, so all 9 studies have the same general pattern (criteria used by the Cochrane Review group). Combining the 9 studies then as described in Methods [21-25], gave 70.26 [95% CI = 34.59–142.72] as the minimum odds ratio that a mantle cell lymphoma contains an ATM mutation (Table 1). The chi^2 ^test value that the pooled odds ratio differs from 1 was 138.30, P < 0.0001. No significant heterogeneity was found by multiple criteria given in Methods but there are only 9 estimates based on 363 people, a comparatively small pooled population.

**Table 1:** Summary of statistical associations for leukemias and lymphomas with BRCA pathway mutations

**Cancer**	**Gene mutation/polymorphism**	**Data from studies with 95% confidence intervals in brackets **[see Additional file 1]	**Combined values from meta-analysis (3 or more studies)**
MCL	ATM	OR = 123.75 [18.84–5056.6] OR = 74.25 [9.34–3203.5] OR = 297 [23.49–1311.9] OR = 83.25 [12.9–3408.7] OR = 81.00 [9.11–3582.4] OR = 25.67 [3.66–1095.9] OR = 57.32 (7.25 – 2490.3) OR = 44 (3.57 – 2186.4) OR = 44 (5.85 – 1898.9)	OR = 70.26 [34.59–142.72]
Lymphomas	NBS1	RR = 1860 [CI = 972.3–3467]	
PLL	ATM	OR = 84.15 [11.43–3549.9] OR = 165.00 [19.51–7007.2] OR = 198 [19.02–8662]	OR = 137.11 [39.68 to 473.76]
ALL	ATM	OR = 16.16 [2.04–724.3] OR = 25.55 [3.14–1144.6] OR = 2.72 [0.86–10.1]	OR = 17.98 [5.37–60.18]
ALL	Fanconi anemia genes	RR = 13.26 [4.11–42.68] RR = 10.76 [3.61–32.03]	
ALL	NBS1	OR = 1.85 [CI = 1.42–2.25]	
Leukemia before age 15	Fanconi anemia genes	RR = 227.4 [170.8–302.1] RR = 127.4 [95.21–170.2]	
CLL	ATM	OR = 46.59 [6.59–1972.5] OR = 15.97 [1.66–762.5] OR = 13.83 [2.11–580.4]	OR = 21.91 [6.57 to 73.09]
CLL	CHEK2 (I157T)	OR = 14.83 [1.85-infinite]	
CLL	BRCA2 (N372H)	OR = 1.45 [1.13–1.86]	
AML	Fanconi anemia genes	RR = 723.4 [385.7–1355.8] RR= 684.8 [371.6–1261.8] RR = 818.2 [2.37–287,689]	RR= 703.35 [363.7–1354.5]
AML	BRCA1	Association discussed in text	

Additional statistical confirmation that MCL is associated with ATM mutation was obtained for the data used. The product-moment linear correlation coefficient was calculated for total experimental samples with MCL vs. experimental samples with ATM mutation. The correlation coefficient value was 0.95 indicating a strong association.

The translocation t(11;14)(q13;q32) is present in almost all mantle cell lymphomas[19,20]. This translocation is consistent with the misrepair of a double strand break. The MCL tumors associated with this translocation correlate with loss of ATM function within the BRCA pathway [Table 1 and Additional file 1, columns 5 and 6].

### ATM mutation: evidence for association with the leukemias T-ALL, B-CLL, and T-PLL

There are very high odds ratios for an association of an ATM deficit not only with MCL, but also with T-ALL, B-CLL, and T-PLL (Table 1). Substantial percentages of any of these diseases associate with misrepair of some double strand break leading to gene rearrangement or deletion within an affected hematopoietic cell lineage. Fusion proteins and/or gene rearrangements have been documented in about 30% of 2367 children with ALL [26], in 11% of B-CLL [27] and in high percentages of atypical CLL with poor prognosis[28]. Table 1 and Additional file 1 also show that ALL and CLL can be associated with any of several BRCA pathway malfunctions. Table 2 summarizes independent information [29-43,15] corroborating high risks for leukemias and lymphomas associated with ATM deficits or with other BRCA pathway abnormalities.

**Table 2:** Independent evidence corroborating associations between a subset of hematologic cancers and mutations in BRCA pathway genes

**Cancer/abnormality**	**Gene mutated**	**Evidence**	**Reference**
Leukemia, NHL	ATM	56 patients with A-T have standardized incidence ratio for leukemia and NHL of 113 (CI = 41–246)	30
Thymomas, lymphoblastic lymphomas	ATM	Atm deficient mice are immunodeficient with a high incidence of thymomas or lymphoblastic lymphomas	30
PLL, ALL and B-CLL	ATM	PLL, ALL and B-CLL tumors have cytogenetic and immunologic similarities to MCL.	30, 31.
T-PLL	ATM	A-T patients have biallelic mutations in ATM and they develop stable clones that progress to T-PLL-like disease	32
B-CLL	ATM	ATM mutant B-CLL tumors have a proven defect in the repair of ionizing radiation induced damage, a function normally mediated by the BRCA pathway. ATM phosphorylates BRCA1 after gamma radiation induced DNA damage.	29
Myeloid leukemias	FANCD1	FANCD1 is the same as BRCA2 and a FANCD1/BRCA2 biallelic defect associates with leukemias that are much more likely to be myeloid than leukemias that develop in those with normal FANCD1/BRCA2. Myeloid leukemias have increased activity of the non-homologous end joining pathway, the less specific alternative to the BRCA pathway.	37
Double strand breaks	Fanconi anemia all types	All Fanconi anemia cells exhibit frequent spontaneous visible chromosome breaks	34–37
Gross chromosomal rearrangements	Brca2	Murine Brca2 is essential to suppress gross chromosomal rearrangements such as translocations after chromosome breakage. Mouse cells with truncated Brca2 accumulate chromosome breaks and aberrant chromatid exchanges.	15, 38
Homologous recombination repair	FANCJ (BRCA1)	BRCA1 interacts with FANCJ. Homologous recombination repair stimulated by double strand breaks is compromised both in FANCJ deficient cells and in cells with BRCA1 mutations that preclude FANCJ interaction.	39, 40
Acute promyelocytic leukemia	BRCA1	BRCA1 was found to co-localize with the promyelocytic leukemia protein (PML) in promyelocytic nuclear bodies that function in heterochromatin remodeling at the G2 phase and PML protein plays an essential role in the organization of the ionizing radiation induced DNA repair complex.	41, 42
Thymoma, T-cell development, chromosomal abnormalities	Brca2	Mice homozygous for a truncating mutation in Brca2 surviving to adulthood die from thymic lymphoma. BRCA2 regulates RAD51 recombinase which is essential in dividing cells. Mice carrying a T-cell specific disruption of the Brca1 gene display markedly impaired T-lymphocyte development and proliferation with increased chromosomal abnormalities.	43, 44

T-PLL is a rare malignant proliferation of post-thymic T-cells, usually with an aberrant T-cell receptor rearrangement that activates oncogenes. Data showing an association between T-PLL and ATM mutation are more limited than data for ATM associations with the other diseases. Nonetheless depending on the study, there was an ATM mutation in 46–66% of 77 tested T-PLL patients [see Additional file 1, columns 5 and 6]. The association is statistically significant but the confidence intervals are broad (Table 1). To verify this association, T-PLL risks were then considered in ataxia-telangiectasia (A-T) patients who inherit biallelic mutations in ATM. A-T patients have a 10% risk for leukemias and lymphomas which is about 100 fold higher than in the general population. In A-T there is a recurrent malignancy similar to T-PLL with a similar course, a similar immunophenotype, and similar cytogenetics [reference [32] and Table 2]. T-PLL occurs at high frequency in A-T families compared to non A-T families[33]. These considerations support the association between ATM mutations and T-PLL.

In some cases, there is no truly reliable way to distinguish somatic from inherited mutations in the BRCA pathway. It is unlikely that this biases the results. The first row in Table 2 gives the risk for some leukemias and lymphomas in A-T patients. Risks for A-T patients are similar to those summarized in Table 1 for ATM mutations in people who do not have hereditary A-T [also see Additional file 1]. Somatic BRCA1 data can also be compared to that for the hereditary disease Fanconi anemia because BRCA1 interacts with the Fanconi protein FANCJ. Therapy related inactivation of BRCA1 (preventing its interaction with FANCJ) causes risks for AML comparable to risks for AML due to hereditary Fanconi anemia (see below).

### Fanconi Anemia genes within the BRCA pathway and early leukemias

I also examined potential associations between hereditary Fanconi anemia gene mutations and risk of hematologic cancers. Results were available from about 80 years of published data in Fanconi anemia databases [34-36]. Data exists from a relatively large number of patients in 3 summary studies. The Fanconi anemia studies each report very large hazard ratios for early leukemias and calculated relative risks are also high [see Additional file 1]. Fanconi anemia patients have a relative risk of 703.35 for AML as calculated by meta-analysis (Table 1). Frequent spontaneous chromosome breaks and gross-chromosomal rearrangements are visible in Fanconi anemia cells (Table 2), consistent with large increases in risk for cancers mediated by chromosome rearrangements. Some data predates the ability to identify individual Fanconi mutations. This merged data was justified for use here because of the close interactions and relationships among Fanconi proteins (Figure 1).

### BRCA1 and BRCA2 genes in the BRCA pathway and AML, leukemias and lymphomas

Independent and unrelated lines of investigation strongly implicate BRCA1 and BRCA2 deficiencies in hematologic cancers. This information is summarized below and there is additional corroborating evidence [15,37-44] in Table 2. BRCA1 deficiency is strongly associated with both de novo- and therapy related AML. 32% (32/112) of primary AML tumors and 75% (16/21) of therapy related AML tumors have reduced BRCA1 gene expression [reference [45] and see Additional file 1]. In chronic myelogenous leukemia (CML) cells, BRCA1 is also down regulated, becoming nearly undetectable in leukemia cells from patients during chronic phase and blast crisis [see Additional file 1]. Deleterious BRCA2 sequence variants are over-represented in cases of T-cell Non-Hodgkins lymphoma (NHL) or CLL [see Additional file 1], consistent with a role for BRCA2 in preventing these diseases.

Relationships and critical interactions exist among BRCA1, BRCA2 and Fanconi anemia proteins(e.g. Fig. 1 and Table 2). Because of connections between Fanconi proteins and leukemias, these relationships further implicate BRCA1/BRCA2 deficits in leukemias. As an example of interactions between Fanconi anemia proteins and BRCA1/2 proteins, the Fanconi anemia protein FANCJ forms an essential complex with BRCA1. This complex brings FANCJ (together with replication protein A) into nuclear foci at the site of DNA damage. FANCJ then unwinds DNA sufficiently so that error-free repair can begin (Table 2). In kindreds who have BRCA2 (FANCD1) mutations on top of another Fanconi anemia mutation, leukemia occurs at a median age of 2.2 years instead of 13.4 years [reference [37] and Additional file 1].

Some epidemiologic studies show increased risks for leukemia/lymphoma[1] in identified BRCA1 or BRCA2 mutation carriers [46,47] and in large populations eligible for mutation testing [48-53]. Family history can be used to determine eligibility for mutation testing and can estimate the likelihood that a BRCA1 or BRCA2 mutation exists within the family[1]. Rauscher et al [54] reported that family history of breast cancer increased risk due to a range of leukemia risk factors that were generally weak or non-existent when considered alone. Combined with a family history of breast cancer, ever-smoking [RR_11 _= 2.4, CI = 1.2–4.8], general solvent exposure (RR_11 _= 1.9, CI = 1.1–3.4), aromatic hydrocarbon exposure (RR_11 _= 3.8, CI = 1.1–14), and diagnostic ionizing radiation exposure (RR_11 _= 2.1, CI = 1.2–3.8) were all associated with increased leukemia incidence. There was no increased incidence associated with any of these exposures in the absence of a family history of breast cancer[54].

### MRE11-Rad50-NBS1: a complex of BRCA pathway genes and lymphomas and leukemias

Abnormalities in a complex containing the BRCA pathway proteins MRE11, Rad50 and NBS1 (MRN complex) also associate with leukemia and lymphoma. Rarely, hypomorphic mutations in MRE11 occur in individuals with "Ataxia-Telangiectasia-Like-Disorder" (ATLD). Lymphocytes from ATLD patients may carry chromosome translocations identical to lymphocytes from A-T patients[55], implying a corresponding predisposition to leukemias and lymphomas. A hypomorphic mutation of the NBS1 gene causes Nijmegan Breakage Syndrome (NBS). Patients in the International NBS study group have enormously elevated risks for lymphoma at ages 1–22 (RR = 1860) [see Additional file 1]. Heterozygotes have lower risks but still appear predisposed to lymphomas and leukemias [see Additional file 1]. Lymphocytes or other cells from NBS patients show increased chromosomal translocations[56].

### CHEK2: a BRCA pathway gene and lymphomas and leukemias

Checkpoint kinase 2 (CHEK2) participates in the BRCA pathway by phosphorylating BRCA1, promoting cell cycle arrest, and activating DNA repair in genetically damaged cells[57]. CHEK2 may also affect risks for hematologic cancers. The CHEK2 variant I157T is significantly associated with CLL [see Additional file 1]. CHEK2 mutations were uncommon in NHL but 9% of these tumors showed either total or near-total absence of the CHEK2 protein[58]. CHEK2 alterations responsible for these low levels occur in a subset of aggressive lymphomas having a relatively high number of chromosomal imbalances[58]. CHEK2 may also have some relationship to promyelocytic leukemia (PML) because CHEK2 phosphorylates the tumor suppressor PML gene protein leading to apoptosis[59] Although the composition of nuclear bodies containing PML varies during the cell cycle, they may also contain other members of the BRCA pathway and participate in double strand break repair[60,61].

## Discussion

Participation of BRCA pathway deficiencies in leukemias and lymphomas can be explained by incorporating features of overlapping theories for how cancers arise. These theories are differentiation-maturation mutations cooperating with proliferation/survival mutations, lineage-addiction or -dependency models, and the existence of "cancer stem cells" arising from an ordered sequence of phenotypically distinct stem-cell and intermediate-precursor populations [62-64]. Applying elements of these current theories helps clarify the present results as discussed below.

Some lymphomas and leukemias are defined by non-random, characteristic gene rearrangements [e.g. [65-68]] but people can have small numbers of cells containing one of these translocations that may not progress to cancer. Thus additional abnormalities are required to create cancer. According to one hypothesis[62], two kinds of gene rearrangements or other mutations cooperate to produce leukemias and lymphomas *(i-ii)*. *i*. Gene rearrangements or other mutations that give a growth and/or survival advantage to malignant cells. *ii*. Gene rearrangements or other mutations that impair differentiation.

Mantle cell lymphoma (MCL) is associated here with a BRCA pathway deficit and this association is consistent with current cancer models. In almost all cases of MCL, there is a characteristic exchange of fragments between chromosome 11 and chromosome 14. The rearrangement occurs within a subset of naive pregerminal center cells in the B-cell lineage. This "MCL translocation" results in the juxtaposition of the BCL1 gene (cyclin D1) and the immunoglobulin heavy chain locus. The MCL translocation causes cyclin D1 to become overexpressed because it comes under the control of the highly active immunoglobulin gene enhancer. Overexpressed cyclin D1 then probably functions as an oncogene by accelerating cell division. This gives a growth advantage to cells containing the rearrangement. In some systems, cancer cell lines are dependent on a cyclin D1 oncogene for survival (oncogene addiction). Abnormal cells here with the cyclin D1 oncogene, have a growth advantage that makes further mutations more likely to accumulate. A BRCA pathway deficit causes an underlying deficiency in error-free repair that increases the number of abnormal cells and adds further to the chances for additional abnormalities. However, the aberrant B-cell lineage may well condition the range of mutations allowed because of embedded differentiation or developmental programs. "Atypical CLL" shares cytogenetic and immunologic features with MCL[32,69]. The same "MCL translocation" between chromosomes 11 and 14 occurs in "atypical CLL." [69], consistent with this macro-genomic alteration being restricted to the B-cell lineage.

AML is another example of a disease associated with a deficit in BRCA-pathway-mediated DNA repair. The hallmark of all AML types is a severe block in myeloid differentiation. In previous sections of this paper, Fanconi anemia patients were shown to have >700 fold increase in combined relative risk for AML (Table 1). In Fanconi anemia, the BRCA pathway deficiency leads to visibly increased numbers of chromosome breaks, gaps, rearrangements, and quadriradii in the presence of DNA damaging agents. This may result from a documented increase in repair by the less specific process of non-homologous end joining.

AML is the generic term for a group of myeloid leukemias that have a clonal expansion of immature myeloid progenitor stages (blasts) in the bone marrow, blood or other tissues. Different categories of AML can depend on a particular mutation event that creates a block in differentiation and the stage within the myeloid lineage when the event occurs. Translocation events, duplications, inversions, or deletions would be favored by BRCA pathway defects and they represent potential ways to create a differentiation block typical of AML. Although other types of mutation also create differentiation blocks in AML, the large increases in relative risk in Fanconi anemia suggests that gene rearrangements are important. In Fanconi anemia, translocations occur at a rate that is at least ten times greater than normal after exposure to ionizing radiation [70].

An example of a translocation capable of creating a differentiation block is the recurring t(3;12)(q26;p13) translocation. In a Fanconi anemia patient, this rearrangement was present in the bone marrow at the time of initial diagnosis of myelodysplastic syndrome (often a precursor of AML). The patient had a normal constitutional karyotype but AML then developed. When acute transformation to AML occurred, cytogenetic analysis found multiple chromosome deletions and rearrangements typical of Fanconi anemia[71]. Fanconi anemia is a rare inherited disease, but the same t(3;12) translocation is sometimes the first and the only cytogenetic abnormality found in AML patients who do not have hereditary Fanconi anemia. This particular rearrangement is thought to predispose to AML as follows[72]. It causes overexpression of the EVI-l gene because EVI-1 becomes driven by the TEL promoter[71,62]. Normally EVI-1 is expressed in early myeloid progenitor cells where it helps determine whether progenitors differentiate or proliferate. Abnormal EVI-1 expression probably contributes to AML by interfering with other genes controlling the commitment to differentiate. These progenitors are designed to proliferate rapidly and then to differentiate. Failure to induce timely differentiation might result in a prolonged proliferation phase favoring the accumulation of additional cooperating events. This places the progenitors at much higher risk for leukemia [63]. A background of hereditary Fanconi anemia would greatly increase chances for gene rearrangements and deletions in progenitors both as initial and as cooperating events.

A variety of gene rearrangements due to misrepaired double strand breaks also occur frequently in other diseases associated here with BRCA pathway deficiencies. In some cases of T-PLL, one gene rearrangement deregulates the expression of the T-cell receptor. Similarly, any of several recurring chromosomal translocations can be detected in substantial numbers of cases of childhood ALL.

Defects in the BRCA pathway increase the risks for a subset of lymphomas and leukemias that are probably associated with gene rearrangements. However a BRCA pathway deficit does not cause the underlying gene rearrangements. The deficit allows more mistakes in double strand break repair, increases the numbers of cells with mistakes and then permits abnormal cells to survive.

In myeloid leukemias, certain sites may associate with up to 40 different gene partners and chromatin structural elements closely associate with such breakpoints [68]. Some of these translocations have prognostic significance. Perhaps certain chromosome regions are selected for these rearrangements because they are more actively transcribed and exposed in a transcription complex[73]. The proximity between neighboring chromosomes may also be an influence.

Some tumors [see Additional file 1] contain evidence that ATM deficiency compromises the BRCA pathway regardless of other pathways involving ATM. Gene fusions or other rearrangements often found in some of these tumors bear witness to a double strand break repaired by error-prone methods. The cancers reported here are thus primarily somatic in origin but the predisposition to misrepair of DNA breaks and chromosomal instability may be inherited. Inactivation of a single gene can increase risks for multiple cancers and inactivation of a different gene in the same pathway may have similar effects [see Additional file 1].

The deficiencies that increase risk for leukemias and lymphomas may well be helpful in understanding other cancers in BRCA1/2 mutation carriers. The involvement of BRCA pathway deficits in a subset of hematologic cancers has implications for surveillance and for therapy in hematologic and perhaps in other cancers. These deficits suggest the need for improved surveillance. They also present a vulnerability that may be exploited during therapy.

Reciprocal translocations and other chromosome rearrangements also occur in breast and in ovarian tumors[74,75]. Comparative genome hybridization has shown that human epithelial breast tumors undergo widespread gains and losses of chromosomes early in their development, correlating well with the presence of complex chromosomal rearrangements [76]. In comparing hematological and epithelial cancers in 2001, Ponder asked "Are there similar mechanisms among the more complex chromosomal changes in epithelial malignancies, or do epithelial cancers have different genetic mechanisms of development?" [77]. The data in the present paper adds the information that the same BRCA pathway can be disabled in both breast and hematological cancers, showing that further consideration of Ponder's question may be very helpful.

## Conclusion

BRCA1 and BRCA2 are critical to prevent breast and ovarian cancers in mutation carriers but the proteins participate in processes that are fundamental for survival in other types of cells. The genetic defect accompanying BRCA pathway deficiencies studied here is a chromosomal misrepair syndrome. This work shows that the pathway containing BRCA1/2 gene products is essential to prevent a group of leukemias and lymphomas. The results may have clinical implications for surveillance and chemotherapy in these and perhaps in other cancers.

## Abbreviations

AML, acute myeloid leukemia; A-T, ataxia-telangiectasia; ATLD, Ataxia-Telangiectasia-Like-Disorder; ATM, ataxia-telangiectasia mutated; B-CLL, (B-cell) chronic lymphocytic leukemia; CHEK2, Checkpoint kinase 2; CI = 95% Confidence Interval; CML, chronic myelogenous leukemia; MCL, mantle cell lymphoma; MRN complex, complex of MRE11, Rad50 and NBS1; NBS, Nijmegan breakage syndrome; NHL, non-Hodgkins lymphoma; OR, odds ratio; PML, promyelocytic leukemia; RR, relative risk; T-ALL, T-cell acute lymphocytic leukemia; T-PLL, T-cell prolymphocytic leukemia.

## Competing interests

The author(s) declare that they have no competing interests.

## Authors' contributions

The author was the sole contributor.

## Pre-publication history

The pre-publication history for this paper can be accessed here:



## Supplementary Material

Additional file 1 Mutation of BRCA pathway components in leukemias and lymphomas. Summaries of case-control, cohort, and basic science research studies that provided numerical, statistical and/or patient data for BRCA pathway gene deficits vs. leukemias and lymphomas. Click here for file
