# Supplementary material for: The BRCA1/2 pathway prevents hematologic cancers in addition to breast and ovarian cancers
Source: BMC Cancer. 2007 Aug 6;7:152. doi: 10.1186/1471-2407-7-152 (PMC1959234; doi:10.1186/1471-2407-7-152)
Supplement: Additional file 1 — Mutation of BRCA pathway components in leukemias and lymphomas. Summaries of case-control, cohort, and basic science research studies that provided numerical, statistical and/or patient data for BRCA pathway gene deficits vs. leukemias and lymphomas. [file 1471-2407-7-152-S1.doc]

### Additional file 1 - Mutation of BRCA pathway components in leukemias and lymphomas

| **Gene / variant** | **One function for gene product consistent with involvement in the pathway** | **Hematologic Cancer / condition** | **Description of patients providing leukemia/lymphoma samples and tests done.** | **Prevalence and percent positive in number of samples tested** | **Results [95% confidence interval]** | **Type of study** | **Reference** |
| --- | --- | --- | --- | --- | --- | --- | --- |
| ATM genomic deletions spanning ATM locus | ATM phosphorylates BRCA1 after gamma radiation induced DNA damage. ATM may function directly in double strand break repair by maintaining DNA ends in repair complexes. | Mantle cell lymphoma | Diagnostic biopsies from 72 cyclin D1 positive MCL cases. Analyzed using RT quantitative PCR | 40/72 (56%) | OR=123.75 [18.84 - 5056.6] | Case - control | 1. Greiner et al. PNAS 2006;103:2352-57. |
| All possible ATM coding and splice site mutations. |  |  | Diagnostic biopsies from 28 Nebraska patients with MCL. 12 of 28 cases had mutations. Of the 12, eight were deleterious and 4 were unclassified. Used microarray based assays with 250,000 oligonucleotides to screen lymphomas | 12/28 (43%) | OR=74.25 [9.34 - 3203.5] | Case - control | 1. Fang et al. PNAS 2003, 100: 5372-77. |
| ATM |  |  | Mutation analyses of 12 cases of sporadic mantle cell lymphoma using RT-PCR and SSCP analysis. None of the patients had a family history of A-T or clinical evidence of A-T | 9/12 (75%) | OR=297 [23.49 - 1311.9] | Case - control | 1. Schaffner et al. PNAS 2000, 97: 2773-6. |
| ATM |  |  | 81 patients with mantle cell lymphoma. Specimens derived from lymph nodes, tonsil, stomach, or conjunctiva. Assayed by FISH. | 37/81 (46%) | OR=83.25 [12.9 - 3408.7] | Case - control | 1. Stilgen-bauer et al. Blood 1999, 94: 3262-64. |
| Missense mutations in the kinase domain and 5 truncating mutations. |  |  | 12 typical and 8 blastoid MCL tumors from Spain. In the 20 MCL tumors CGH found 9 deletions of 11q, all with low or absent ATM protein expression. | 9/20 (45%) | OR=81.00 [9.11 - 3582.4] | Case - control | 1. Camacho et al. Blood 2002, 99:238-44. |

| All tumors had a demonstrated t(11;14) (q13;32) translocation. |  |  | 68 patients newly diagnosed with MCL, median age 70 range 46-86. | 11/30 (37%) | OR=25.67[3.66-1095.9] | Case - control | 1. Jarosova et al. Leuk Lymphoma 2004, 45:1835-46. |
| --- | --- | --- | --- | --- | --- | --- | --- |
| ATM T(11;14) |  |  | 30 mantle cell lymphoma patients from Czechoslovakia assayed by CGH at time of diagnosis. T(11;14) found in 20. 20 cases were defined as typical and 10 as blastoid. 8 women and 22 men with median age 66.5 ranging from 39-80. Assay was mainly by CGH with array CGH in 2 cases. | 14/68 (21%) | OR=57.32 (7.25 - 2490.3) | Case - control | 1. Rubio-Moscardo et al Blood 2005, 105: 4445-6. |
| ATM Loss of 11q23 region |  |  | 13 patients with Mantle cell lymphoma assayed by CGH. A subgroup of patients randomly selected from 94 with B-cell NHL (ages 31-86, median 57). Assay of loss of 11q regions was by CGH. Loss of ATM gene region at 11q21-23.1 was significantly associated with disease (p<=.03) | 4/13 (31%) | OR=44 (3.57 - 2186.4) | Case - control | 1. Stokke et al. Brit J Cancer 2001, 85:1900-13. |
| ATM deletion on chromosome 11q mapped to 11q22-23 |  |  | 39 patients (26 men, 13 women) age 47-87; mean 65 y with MCL positive for t(11;14). Assay was by CGH, FISH or by both. | 12/39 (31%) | OR=44 (5.85 - 1898.9) | Case - control | 1. Bentz et al. Genes Chromosomes Cancer 2000, 27: 285-294. |
| ATM Missense mutations clustered in the kinase domain. |  | T-cell PLL | 37 patients from England or Sweden with sporadic T-cell PLL assayed by exon scanning SSCP. Pattern of missense differs from classical A-T where truncating mutations predominate. | 17/37 (46%) | OR=84.15 [11.43 - 3549.9] | Case - control | 1. Vorechovsky et al. Nat Genet 1997, 17:96. |
| ATM Deletion of 11q22.3-23.1 |  | T-cell PLL | 24 T-PLL in patients median age 66 y with a range of 43-79 showing no signs of A-T. No non malignant specimens available to assess germ line status. The chromosome region 11q22.3-23.1 containing ATM was deleted in 15 of 24 T-PLL tumors studied. | 15/24 (63%) | OR=165.00 [19.51 - 7007.2] | Case - control | 1. Stilgen-bauer et al. Nature Med 1997, 3:1155-9. |
| Deletion of ATM gene |  | T-cell PLL | Paired leukemic and non leukemic cells from 15 T-PLL patients. LOH in region 11q22-23 | 10/15  (66%) | OR= 198 [19.02 - 8662] | Case-control | 1. Stoppa-Lyonnet et al Blood 1998, 91:3920-26 |
| ATM coding exon mutation |  | T-ALL | 57 childhood sporadic ALL cases from Switzerland: 26 B-precursor ALL and 31 T-ALL. 8 patients had alterations of probable biologic significance. 66 coding exons of ATM analyzed by DHPLC + PCR. In 2 patients alterations were present in the germline. | 8/57 (14%) | OR=16.16 [2.04 - 724.3] | Case -control | 1. Gumy-Pause, et al. Hum Mutat Mutation In Brief #611 Online 2003. |
| ATM three truncating changes and missense variants |  | T-ALL | 39 pediatric T-cell ALL. 8 T-cell samples had one of 3 truncating changes or 3 missense variants. A-T carriers were 4.9-12.9 times more frequent than in the general population. | 8/39 (21%) | OR=25.55 [3.14 - 1144.6] | Case -control | 1. Liber-zon et al. Genes Chromosomes Cancer 2004,39: 161-6. |
| ATM Examined 20 different ATM alterations |  | T-ALL | DHPLC in T-lineage ALL from Dutch and German children ages 1.1-16.7 years. Substitutions found in 16/103 T-ALL vs 51/99 controls. Five coding alterations occurred more frequently in T-ALL children than in controls (13% vs. 5%). The alterations are associated with a high white count and unfavorable outcome. | 13/103 (13%) | 2.72 [0.86 - 10.1] Controls were 5 coding alterations in 99 samples. | Case -control | 1. Meier et al. Leukemia 2005, 19:1887-95. |
| ATM Mutations observed were loss of one allele with mutation of the second; |  | CLL | 50 unselected patients with classical CD5+ and CD23+ B-CLL with median age 72.5 (range of 39-90). Follow-up was for at least 2 years. 2 mutations were found in 5 tumors; 5 tumors had a single point mutation with 2 of these leading to premature truncation. Only about 70% of ATM mutations were detected. Mutations were restricted to pre-germinal B- cells. | 16/50 (32%) | 46.59 [6.59 - 1972.5] | Case -control | 1. Stan-kovic et al. Blood 2002,99:300-309. |
| LOH at ATM locus and protein level |  | CLL | 5 of 36 cases showed LOH at ATM locus with expression of protein reduced in about 34% | 5/36 (14%) | 15.97 [1.66 - 762.5] | Case -control | 1. Star-ostik et al. Cancer Res 1998 58: 4552-7. |
| ATM mutations including 11q deletions |  | CLL | 155 B-CLL tumors analyzed and 12% had ATM mutations, usually present at diagnosis. Patients with these mutations had statistically significant reduced disease free survival. | 19/155 (13%) | 13.83 [2.11 - 580.4] | Case -control | 1. Austen et al. Blood 2005, 106: 3175-82 |
| ATM F858L (SNP) |  | CLL | 992 cases and 2707 controls assayed for non-synonymous amino acid altering polymorphism |  | OR=2.28 [1.53-3.40] | Case -control | 1. Rudd Blood online March 30, 2006. |
| ATM P1054R |  | CLL | 992 cases and 2707 controls assayed for amino acid altering polymorphism |  | OR=1.68 [1.25-2.28] | Case -control |  |
| Fanconi anemia genes | Complex of Fanconi anemia proteins triggers the ubiquitylation of FANCD2, which co-localizes with BRCA1 at sites of DNA damage. FANCJ (BRIP1 /BACH1) is a helicase that interacts with BRCA1. Functional Fanconi anemia proteins are essential to prevent spontaneous chromosome breaks. | AML | 755 North American Fanconi anemia patients followed for 20 years. International Fanconi anemia registry established 1982 at Rockefeller University. | 33% have a hema-tologic malig-nancy by age 40 | RR=723.4 [385.7-1355.8] | Cohort | 1. Kutler et al. Blood 2003, 101:1249-56 |
| Fanconi anemia genes |  | AML | All available cases from 1927-2001. 1301 cases in the literature | 37% have leukemia by age 29. 50% have MDS by age 43. | RR=684.8 [371.6-1261.8] | Cohort | 1. Alter B. Blood 2003, 97:425-440. |
| Fanconi anemia genes |  | AML | 145 North American patients studied retrospectively | 9 cases of leukemia observed vs. 0.011 expected | RR=818.2 [2.37-287,689] | Cohort | 1. Rosen-berg et al . Blood 2004, 104:350-55 |
| Fanconi anemia genes |  | Leukemia before age 15 | 755 North American Fanconi anemia patients followed for 20 years. International Fanconi anemia registry established 1982 at Rockefeller University. | 33% have a hema-tologic malig-nancy by age 40 | RR= 227.4 [170.8-302.1] | Cohort | ***20.*** Kutler et al. Blood 2003, 101:1249-56 |
| Fanconi anemia genes |  | Leukemia before age 15 | All available cases from 1927-2001. 1301 cases in the literature | 37% have leukemia by age 29. 50% have MDS by age 43. | RR=127.4 [95.21-170.2] | Cohort | ***21.*** Alter Blood 2003, 97:425-440 |
| Fanconi anemia genes |  | ALL | 755 North American Fanconi anemia patients followed for 20 years. Rockefeller International Fanconi anemia registry established 1982 | 33% have a hema-tologic malig-nancy by age 40 | RR=13.26 [4.11-42.68] | Cohort | ***20.*** Kutler et al. Blood 2003, 101:1249-56 |
| Fanconi anemia genes |  | ALL | All available cases from 1927-2001. 1301 cases in the literature | 37% have leukemia by age 29.  50% have MDS by age 43. | RR=10.76 [3.61-32.03] | Cohort | ***21.*** Alter Blood 2003, 97:425-440 |
| BRCA1 | Role in homologous recombination DNA repair but many other functions. Interacts with FANCJ and accompanies PML. | AML primary | mRNA assayed in blood granulocytes, monocytes, lymphocytes and mononuclear cells | 32/112 cases of primary AML had a hyper-methylated BRCA1 promoter.  51/133 (38%) cases of therapy related AML had a hyper-methylated BRCA1 promoter | Reduced or absent BRCA1 expression due to promoter hyper-methylation is frequent in primary and therapy related AML. | Case -control | 1. Scar-docci et al Brit J Cancer 2006, 95: 1108-13 |
|  |  | CML | Primary leukemia cells from CML patients, human and murine cell lines. |  | Expression of BCR-ABL fusion protein accompanies down regulation of BRCA1 protein so that it is nearly undetectable in CML patients. | Basic | 1. Deutsch et al Blood 2003, 101: 4583-88. |
| BRCA2 (FANC-D1) | BRCA2 is essential for repair by homologous recombination but not needed for repair by non-homologous end joining.  Identical to FANCD1. Maintains genomic stability preventing gross chromosomal translocations. Regulates RAD51 recombinase which is essential for dividing cells. | AML | 7 patients in 5 kindreds | 5 cases |  | Cohort | 1. Wagner et al Blood 2004, 103: 3226-3229. |
| BRCA2 |  | ALL | 7 patients from 5 kindreds with Fanconi anemia. Leukemia occurred at a median age of 2.2 years, in contrast to 13.4 years for all other patients with Fanconi anemia. | 2 cases |  | Cohort |  |
| BRCA2 N372H (SNP) |  | CLL | 992 cases and 2707 controls assayed for non-synonymous single nucleotide polymorphisms |  | OR=1.45 [1.13-1.86] | Case -control | ***19****.* Rudd et al Blood online March 30, 2006. |
| BRCA2 Asn289His |  | T-cell NHL | 35 controls and 7 cases | 7 cases | OR=3.97[1.60-9.90] | Case -control | ***26.*** Shen et al Hum Genet 2006,119:659-68 |
| NBS1 | Forms complex with MRE11 and Rad50 involved in recombination repair of double strand breaks. Complex senses DNA damage and activates ATM. | Lymphomas | 55 patients with NBS in the International Nijmegan breakage study group. 22 patients developed malignancy and 16 of these were lymphomas. Ages 1-22 | 16/55 vs. 6.1/1,000,000 for ages 0-14, incidence of NHL in 1795 children or 15.6 per 100,000 person years for all lym-phomas from 12 SEER registries | RR=1860 [972.3-3467] | Cohort | 1. Inter-national NBS Study Group 2000, Arch Dis Child 2000, 82:400-6 2. Morton et al Blood 107, 1:266-76 |
|  |  | ALL, AML | Remission DNA samples from 321 children with primary leukemia and lymphoma tested for R215W variant in NBS1. | 4 leukemia patients (2-b-ALL, 1 T-ALL, and 1 AML and had this variant. | OR=2.6 but non-significant | Case -control | 1. Taylor et al. CancerRes 2003, 63:6563-4. |
|  |  | ALL | Heterozygotes for NBS1 founder mutation 657del5 found in 3 of 270 patients with sporadic lymphoid malignancies compared to 6984 matched controls. | 3/270 patients | OR=1.85 [CI=1.42-2.25] | Case -control | 1. Chrzanowska et al. Int J. Cancer 2005, 118:1269-74.. |
|  |  | NHL |  | 2/212 patients | OR=1.57 [1.21-2.25] |  |  |
| MRE11 | Part of a conserved complex with RAD50 and NBS1. Colocalizes with BRCA1 after DNA damage. Complex is essential for double strand break repair by homologous recombination, perhaps acting as a damage sensor, phosphorylating ATM. | Chromo-some trans-locations in ATLD and similarities to A-T. | Very rare condition affecting a few dozen known patients world-wide. Cells have chromosome instability and increased sensitivity to ionizing radiation. |  |  | Cohort | 1. Stewart et al Cell 1999, 99:577-87 |
| CHEK2 I157T | Chek2 is a downstream effector of ATM CHEK2 directly phosphorylates BRCA1 perhaps modifying BRCA1 intracellular location. | CLL | 992 cases and 2707 controls assayed for amino acid altering polymorphisms |  | OR=14.83 [1.85-infinite] | Case -control | ***19****.* Rudd et al Blood online March 30, 2006. |
